# IHC-based subcellular quantification provides new insights into prognostic relevance of FLIP and procaspase-8 in non-small-cell lung cancer

**DOI:** 10.1038/cddiscovery.2017.50

**Published:** 2017-08-14

**Authors:** Ryan A Hutchinson, Helen G Coleman, Kathy Gately, Vincent Young, Siobhan Nicholson, Robert Cummins, Elaine Kay, Sean O Hynes, Philip D Dunne, Seedevi Senevirathne, Peter W Hamilton, Darragh G McArt, Daniel B Longley

**Affiliations:** 1 Centre for Cancer Research and Cell Biology, Queen’s University Belfast, Belfast, Northern Ireland, UK; 2 Centre for Public Health, Queen’s University Belfast, Belfast, Northern Ireland, UK; 3 Department of Cardiothoracic Surgery, St James’s Hospital, Dublin, Ireland; 4 Department of Pathology, Education and Research Centre, Royal College of Surgeons of Ireland, Beaumont Hospital, Dublin, Ireland

## Abstract

In this study, we developed an image analysis algorithm for quantification of two potential apoptotic biomarkers in non-small-cell lung cancer (NSCLC): FLIP and procaspase-8. Immunohistochemical expression of FLIP and procaspase-8 in 184 NSCLC tumors were assessed. Individual patient cores were segmented and classified as tumor and stroma using the Definiens Tissue Studio. Subsequently, chromogenic expression of each biomarker was measured separately in the nucleus and cytoplasm and reported as a quantitative histological score. The software package pROC was applied to define biomarker thresholds. Cox proportional hazards analysis was applied to generate hazard ratios (HRs) and associated 95% CI for survival. High cytoplasmic expression of tumoral (but not stromal) FLIP was associated with a 2.5-fold increased risk of death in lung adenocarcinoma patients, even when adjusted for known confounders (HR 2.47, 95% CI 1.14–5.35). Neither nuclear nor cytoplasmic tumoral procaspase-8 expression was associated with overall survival in lung adenocarcinoma patients; however, there was a significant trend (*P* for trend=0.03) for patients with adenocarcinomas with both high cytoplasmic FLIP and high cytoplasmic procaspase-8 to have a multiplicative increased risk of death. Notably, high stromal nuclear procaspase-8 expression was associated with a reduced risk of death in lung adenocarcinoma patients (adjusted HR 0.31, 95% CI 0.15–0.66). On further examination, the cells with high nuclear procaspase-8 were found to be of lymphoid origin, suggesting that the better prognosis of patients with tumors with high stromal nuclear procaspase-8 is related to immune infiltration, a known favorable prognostic factor. No significant associations were detected in analysis of lung squamous cell carcinoma patients. Our results suggest that cytoplasmic expression of FLIP in the tumor and nuclear expression of procaspase-8 in the stroma are prognostically relevant in non-small-cell adenocarcinomas but not in squamous cell carcinomas of the lung.

## Introduction

Non-small-cell lung cancer (NSCLC) accounts for approximately 85% of all lung cancer diagnoses and is the most common cause of death from cancer globally.^1–3^ The AJCC/UICC-TNM system remains the gold standard for assessing patient prognosis; however, it gives no prediction for treatment benefit. NSCLC has one of the lowest 5-year survival rates of all cancers; therefore, the discovery of new biomarkers that can be used as predictive, prognostic, therapeutic or diagnostic tools is of increasing importance. Over the years, numerous molecular pathways and processes have been identified that underpin lung cancer pathogenesis.^4–9^ One of the most investigated mechanisms in cancer research has been apoptosis, which frequently becomes dysregulated and contributes significantly to both disease progression and drug resistance.^10,11^ Apoptosis is an essential physiological mechanism that regulates cell proliferation and tissue homeostasis, which is why its dysregulation is one of the hallmarks of cancer.

Fas-associated death domain-like interleukin 1*β*-converting enzyme (FLICE)-inhibitory protein (FLIP) is a major apoptosis inhibitor, and its overexpression inhibits the activation of caspase-8 (FLICE)-mediated apoptosis.^12^ Clinical analyses have revealed that FLIP is frequently overexpressed in many cancers, including NSCLC.^12^ In addition, previous studies have shown that FLIP expression correlates with tumoral metastatic potential, which may be related to its ability to inhibit anoikis, a form of cell death induced following detachment of anchorage-dependent cells from their extracellular matrix.^13,14^ Procaspase-8 is an initiator caspase that has been found to be inactivated through epigenetic or genetic mechanisms in a subset of human cancers, such as small-cell lung cancer (SCLC); however, the majority of solid tumors appear to retain procaspase-8 expression or even have elevated procaspase-8 expression compared with adjacent normal tissues.^15^ Although the reasons for this are unclear, retention or elevation of procaspase-8 levels may be selected for during tumorigenesis or therapeutic intervention, because in complex with the long FLIP splice form (FLIP_L_), procaspase-8 suppresses another form of cell death termed programmed necrosis or ‘necroptosis’.^16,17^ Thus a delicate interplay exists between FLIP and procaspase-8 that is critical for regulating at least two major forms of cell death and that can therefore have a major impact on disease progression and response to a range of anticancer therapeutics. For example, we have previously demonstrated in several cancers including NSCLC that downregulating FLIP enhances chemotherapy-induced apoptosis in a caspase-8 dependent manner, whereas overexpression of FLIP blocks caspase-8-mediated apoptosis.^15,18,19^

NSCLC consists of several histological subtypes, of which adenocarcinoma and squamous cell carcinoma predominate.^20^ In this study, we used a digital pathology approach for measuring FLIP and procaspase-8 expression in the cytoplasmic and nuclear compartments of NSCLC tissues. Because of the increasingly recognized importance of the tumor microenvironment, we also determined the expression of both proteins in the adjacent stromal regions. Our findings indicate that high cytoplasmic FLIP expression in tumor cells is an important determinant of patient prognosis for non-small-cell adenocarcinoma but not for squamous carcinoma. Moreover, we unexpectedly found that high nuclear expression of procaspase-8 in the stromal compartment is strongly associated with improved prognosis.

## Results

### Automated quantification of FLIP and procaspase-8

A tissue microarray (TMA) was constructed using normal and tumor tissues from a cohort of 184 NSCLC patients and immunohistochemically assessed for the expression of FLIP and procaspase-8.^21^ To achieve both automated quantification and obtain a continuous range of FLIP and procaspase-8 staining intensities, the TMA images were imported into Definiens Tissue Studio (Definiens AG, Munich, Germany), an image analysis software platform that supports the design, development and application of new image analysis algorithms. For each of the biomarkers being investigated in this study, a novel image analysis method was created using the following steps:

#### (1) Automated tumor identification

**Figure 1:**
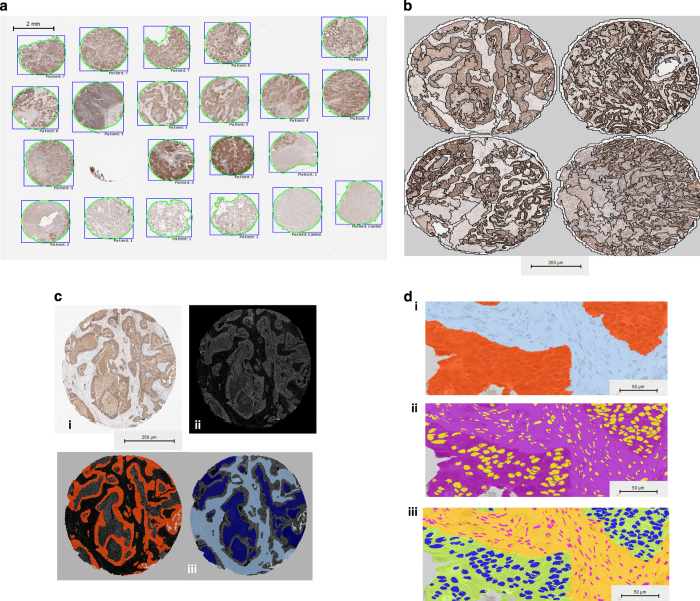
(**a**) Each patient’s core was individually de-arrayed from the digital slide, and patient metadata was assigned to each core for future data mining and clinical correlations. (**b**) To develop an automated tumor stroma classifier, each core was segmented into super-pixels using a multi-resolution segmentation algorithm. (**c**) Subpanel (i) shows an individual patient core, which in subpanel (ii) was subjected to a stain isolation algorithm that was used to separate the image into positive and negative staining. In subpanel (iii), super-pixels were classified as either tumor (orange) or stroma (sky blue); regions of necrosis (deep blue) were also identified. (**d**) Following tumor and stroma assignment in subpanel (i), regions were resegmented and a generic nucleus detection algorithm was applied, shown in subpanel (ii) with nuclei of various morphologies coloured yellow. Subsequently, as depicted in subpanel (iii), classification of tumor nuclei (blue) within the tumor matrix (green) and stromal nuclei (pink) within the stromal matrix (yellow) was performed.

Once imported into Definiens, a custom grid was defined to match the layout of the TMA. Patient clinical metadata were linked to each core, and the cores were then digitally ‘de-arrayed’ to generate individual cores and assign array coordinates to match the layout of the original TMA (Figure 1a). Twelve of the de-arrayed cores, which demonstrated a range of biomarker expression positivity, were used in a training set (Figure 1a). First, a threshold to distinguish between background and whitespace was defined using a combination of thresholds, which included a homogeneity threshold that segmented the image into homogeneous and non-homogeneous regions. These segmented regions were then classified as background and tissue core; additionally, a minimum area parameter that classified artifacts as part of the background was included. Each individual tissue core was segmented into super-pixels using a multi-resolution segmentation algorithm (Figure 1b), which partitioned the image into spectrally coherent regions. Within each super-pixel, the brown chromogen intensity, hematoxylin intensity, area and shape as well as customized arithmetic (ratios of hematoxylin to brown chromogen intensity) and temporo-spatial features (distance of tumor regions to stroma and whitespace) were measured. Using these features, in combination with a machine learning approach, each super-pixel was classified as either tumor or stroma (Figure 1c). From each of these classified super-pixels, nuclei, cytoplasm and cell boundaries were identified (Figure 1d). This subsequently allowed the separate quantitative analysis of protein expression in both the tumor and stromal compartments. Before the algorithm was applied across the complete patient cohort, an experienced Pathologist confirmed that the identification and classification of regions was accurate in an independent training set.

#### (2) Detection and classification of tumoral and stromal nuclei

A stain deconvolution algorithm was used to separate the DAB chromogen stain and the hematoxylin counterstain in all tissue cores. Using super-pixels to define tissue compartments, thresholds were identified to distinguish between positive and negative nuclear expression of each protein using the image object information table within the software. Nuclei were segmented on a nucleus-by-nucleus basis, which enabled visualization of multi-parametric features of each individual nucleus (Figure 1d). The image object table enables the identified nuclei to be sorted by features of interest; in this case, morphology, hematoxylin intensity and brown chromogen intensity were used. From these features, brown chromogen intensity and hematoxylin intensity thresholds were determined, and we used these to segment and identify both positive and negative nuclei within the tumor and positive and negative nuclei within stromal regions. Using this information, a nuclei filter was applied, which enabled us to remove nuclei that did not match the morphological and staining criteria. Nuclei were identified based on measures of their size, sphericity, optical density and level of overlap. A nucleus classification module was added to the algorithm, and using the image object information table, the minimum and maximum brown chromogen intensity of each individual positive nucleus was determined. Nuclei detected using the hematoxylin threshold remained negative when the chromogenically positive nuclei were classified.

#### (3) Identification of cell boundaries and cytoplasmic compartments

In order to be able to assess the cytoplasmic intensity of the proteins, cell simulation was used to ‘grow’ a cytoplasmic compartment for each cell from the detected nucleus. Using the training set, a ‘region of growth’ of 4 *μ*m was identified as the optimal threshold for cell border generation; the growth and morphology of these generated cells were determined through the visualization of a ‘cell mask’. From this cell simulation module, the area between the identified nucleus and cell boundary was classified as cytoplasm. The final stage in the algorithm development was to classify cytoplasmic expression of each biomarker within the regions of interest. The brown chromogen intensity of each individual cytoplasmic region was used to determine the threshold for cytoplasmic expression of each biomarker.

#### (4) Determination of a compartment-specific biomarker histological score (H-score)

From the segmented and classified tumoral and stromal cells, the expression was quantified in each compartment, and using brown chromogen intensity and hematoxylin intensity thresholds, a H-score was calculated for nuclear and cytoplasmic FLIP and procaspase-8 using the formula: (1×percentage of low brown chromogen intensity-positive cells)+(2×percentage of medium brown chromogen intensity-positive cells)+(3×percentage of high brown chromogen intensity-positive cells) based on the quantitative data generated from the image analysis algorithm. Utilizing Definiens Image Miner 2.0 (Definiens AG, Munich, Germany), we created a customized data repository, which linked the clinicopathological data with the quantitative image analysis data. When the algorithm was applied across each TMA, a quality control step was adopted to ensure the algorithm was performing robustly and was not specific to the training set. Random cores were selected from each analyzed TMA, and the tumor identification method, cellular segmentation and quantification were checked by two of the study investigators (RAH and PWH).

### High cytoplasmic FLIP in tumor cells is an adverse prognostic marker in adenocarcinoma

**Figure 2:**
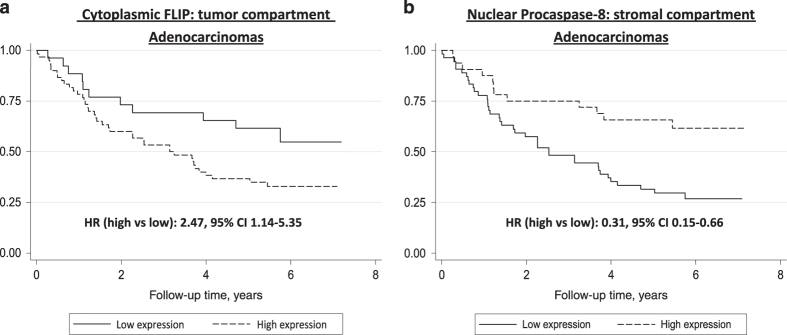
Kaplan–Meier graphs showing overall survival in lung adenocarcinoma patients according to: (**a**) tumor cytoplasmic FLIP expression; and (**b**) stromal nuclear procaspase-8 expression. CI, confidence interval; HR, hazard ratio.

Table 2 shows the association between overall survival of lung adenocarcinoma patients and the expression of tumoral and stromal FLIP and procaspase-8 expression within the nucleus and cytoplasm. High cytoplasmic expression of tumoral FLIP (H-score >172.5) was associated with a two-fold increased risk of death in lung adenocarcinoma patients, which strengthened further when adjusted for known confounders (HR 2.47, 95% CI 1.14–5.35; Table 2; Figure 2a). High tumoral FLIP nuclear expression (H-score>105) was also associated with an increased risk of death, although this did not achieve statistical significance. In contrast, higher stromal FLIP expression in the nucleus and cytoplasm was associated with non-significant decreased risks of death.

### High nuclear caspase-8 in stroma is a favorable prognostic marker in adenocarcinoma

Neither nuclear nor cytoplasmic tumoral procaspase-8 expression was associated with overall survival in lung adenocarcinoma patients. Unexpectedly, however, high stromal nuclear procaspase-8 expression (H-score>90.5) was associated with a reduced risk of death in lung adenocarcinoma patients, which was significant when considering nuclear expression (adjusted HR 0.31, 95% CI 0.15–0.66; Table 2, Figure 2b). Results remained largely similar in sensitivity analysis excluding patients who died in the first 6 months of follow-up (data not shown). There was also a similar trend with high stromal cytoplasmic procaspase-8 expression (H-score>139.5), although this did not reach significance (adjusted HR 0.51, 95% CI 0.23–1.11). Under further pathological examination (RAH, SH, PWH), the stromal cells expressing high procaspase-8 had clear lymphoid cell characteristics, suggesting that high stromal procaspase-8 expression identifies tumors with high degree of immune infiltration.

Notably, no significant associations between overall survival of lung squamous cell carcinoma patients and expression levels of either protein in the nucleus or cytoplasm of tumor or stroma were observed (Table 3), suggesting that FLIP and procaspase-8 are more relevant in non-small-cell lung adenocarcinomas.

### FLIP/procaspase-8 correlation and combined prognostic relevance

**Figure 3:**
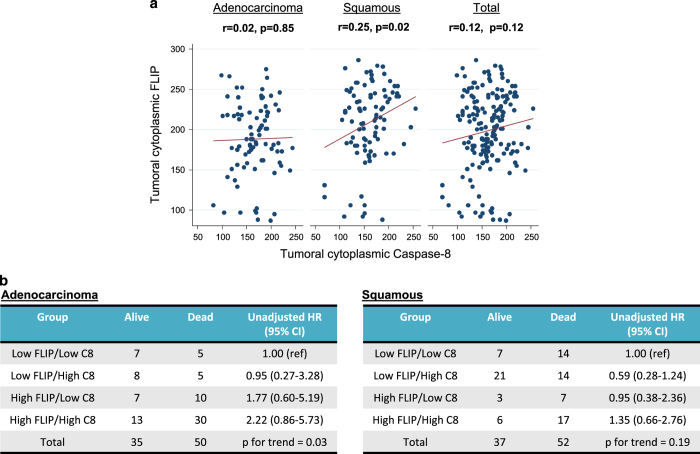
(**a**) Scatterplot showing correlations between tumor cytoplasm expression of FLIP and procaspase-8. Pearson’s correlation coefficients were generated in the adenocarcinoma and squamous cell carcinoma subtypes separately and in the entire study cohort. (**b**) Unadjusted hazard ratios (HRs) and 95% confidence intervals (CIs) for risk of death in lung adenocarcinoma and squamous cell carcinoma patients according to combined categories of high and low FLIP and procaspase-8 cytoplasm expression levels. Cutoffs for high and low expression are outlined in Tables 2 and 3.

Given that FLIP is a key modulator of procaspase-8 activation in the cytoplasm and that these proteins are transcribed from the same genetic locus (*2q33-q34*), we examined whether there was a correlation between their expression at the protein level. In the overall patient cohort, there was no significant correlation between expression of the two proteins; however, in the squamous (but not the adenocarcinoma) subset, a significant positive correlation was observed (*r*=0.25, *P*=0.02; Figure 3a). We further determined whether combined assessment of cytoplasmic FLIP and caspase-8 expression had prognostic relevance. Although, no significant associations between individual combined categories and death were observed, there was a significant trend (*P* for trend=0.03) for patients with adenocarcinomas with both high cytoplasmic FLIP and high cytoplasmic procaspase-8 having a multiplicative increased risk of death; however, no such association was observed in the squamous histology subgroup (Figure 3b).

## Discussion

Procaspase-8 and its endogenous inhibitor FLIP are key regulators of the extrinsic apoptotic pathway activated by cell surface death receptors and are also key regulators of cell death induced by cytoplasmic complexes, such as the ripoptosome.^12,22^ We have previously reported that FLIP and procaspase-8 expression are key determinants of response to chemotherapy and ionizing radiation in preclinical models of NSCLC.^15,23^ We therefore used a digital pathology approach to evaluate nuclear and cytoplasmic FLIP and procaspase-8 expression in a cohort of NSCLCs containing both adenocarcinoma and squamous cell carcinoma histological subtypes.

By defining H-score cutoffs using the ROC approach and by performing multivariate analyses that demonstrate that cytoplasmic FLIP expression is an independent prognostic factor in adenocarcinoma patients but not in squamous cell carcinoma patients, this study refines our previous findings that high cytoplasmic FLIP expression is a biomarker of poor prognosis in NSCLC.^21^ Of note, nuclear FLIP expression was not significantly prognostic in adenocarcinoma patients, highlighting the value of analyzing protein expression at a subcellular level. Our previous analysis of this patient cohort using a generic method for scoring procaspase-8 failed to identify the prognostic significance of nuclear procaspase-8 in the stromal compartment that was revealed in this study, further underlining the potential utility of this methodological approach.

As a major inhibitor of cell death induced by death ligands expressed by immune effector cells, high FLIP expression has been shown to promote immune escape of tumors in mouse models.^24^ Hence, it is plausible that FLIP overexpression is selected for during tumorigenesis to enable tumor cells evade immune elimination via apoptosis. Also, as noted above, FLIP confers resistance to chemotherapy- and ionizing radiation-induced apoptosis,^15,23^ which is again consistent with the association that we have observed between high FLIP expression and poor prognosis. Unlike SCLCs, which frequently lose procaspase-8 expression,^25^ NSCLCs tend to maintain or even overexpress procaspase-8.^15^ This may be driven by the necessity to suppress another form of cell death, necroptosis; procaspase-8 and FLIP_L_ form an enzymatic complex, which although lacking the ability to promote apoptosis, can cleave receptor-interacting kinase-1, a key mediator of necroptosis.^22^

We have previously demonstrated that depletion of FLIP using histone deacetylase (HDAC) inhibitors, such as Entinostat, which has recently been FDA approved for the treatment of aromatase inhibitor-resistant, estrogen receptor-positive breast cancer, results in caspase-8-dependent apoptosis induction and enhances the antitumor effects of chemotherapeutic agents, ionizing radiation and TRAIL.^15,21,23^ The results presented here suggest that HDAC inhibitors or other agents that target FLIP could potentially be targeted against poor prognostic lung adenocarcinomas expressing high cytoplasmic levels of FLIP. Similarly, tumors with low cytoplasmic FLIP expression may be effectively targeted with TRAIL receptor-targeted therapeutics, the second generation of which are in preclinical and clinical development.^26,27^ Another emerging targeted therapy are the IAP antagonists (also referred to as SMAC mimetics),^28^ which we have shown are dependent on FLIP for their efficacy;^29^ thus these agents may also be most effective in NSCLCs with low cytoplasmic FLIP expression.

Given their close biological relationship, we assessed the correlation between FLIP and procaspase-8 protein expression and their combined prognostic relevance. Although cytoplasmic expression of FLIP and procaspase-8 were more strongly correlated in squamous cell carcinomas, the combined expression of these proteins was more prognostic in adenocarcinoma. Of note, high nuclear procaspase-8 expression was apparent in cells of lymphoid morphology in the stroma of tumors of adenocarcinoma patients with good prognosis. Although predominantly expressed in the cytoplasm, sumoylated caspase-8 translocates to the nucleus.^30^ Moreover, caspase-8 has been reported to have a broad role in the activation of immune cells; for example, caspase-8 has been shown to be essential for T-cell proliferation, as specific deletion of the gene encoding caspase-8 (*CASP8*) in T cells results in depletion of the peripheral T-cell population, thereby impairing immune responses.^12,31–33^ It is possible that lymphoid cells with high nuclear caspase-8 promote antitumor immunity, which could explain the association between high nuclear procaspase-8 in the stromal compartment and good prognosis. Indeed, high density of CD4 and CD8 positive T cells in cancer stroma has been correlated with longer survival times in NSCLC patients.^34,35^

In conclusion, this study demonstrates the potential of digital imaging to quantify immunohistochemical expression of putative biomarkers in tumor and stromal compartments at a subcellular level to generate a quantified output, in this case a region-specific H-score. The methodology described herein could ultimately be developed to stratify adenocarcinoma NSCLC patients for therapies that are dependent on FLIP and procaspase-8 expression for their activity.[Table tbl1]

## Subjects and methods

### Clinicopathological details

The tissue samples used in this study are from a cohort of 184 NSCLC patients (86 adenocarcinomas, 9 other histologies and 89 squamous cell carcinomas) of various stages from St James’ Hospital, Dublin, Ireland. As shown in Table 1, 59 and 58% of lung adenocarcinoma and squamous cell carcinoma patients had died by the end of the follow-up period (median 3.8 years, range 0.01–7.4 years), respectively. Nine lung adenocarcinoma and 13 squamous cell carcinoma patients died within the first 6 months, and 16 patients of each histological subtype died within the first year of follow-up. The median age at surgery for lung cancer patients was 66 years. Slightly more males than females were included, particularly in the squamous cell carcinoma group (70.8 *versus* 29.2%). The majority of lung cancer patients were current or former smokers. The majority of tumors represented early pathological stage (52–57% Stage I), tumor grade II (59–62%) and a median tumor size of approximately 4 cm in both histological subgroups. Most lung adenocarcinoma patients underwent lobectomy (80.2%), with the remainder requiring more extensive surgical resection. The majority of lung squamous cell carcinoma patients also underwent lobectomy (67.4%); however, a greater proportion of this patient group required pneumonectomy compared with adenocarcinoma patients (28.1 *versus* 8.1%).

### Immunohistochemical detection methods

Anti-caspase-8 (11B6, Leica Microsystems, Milton Keynes, UK) and anti-FLIP (G-11, Santa Cruz, Heidelberg, Germany) antibodies were used as previously described, with hematoxylin used as a counterstain.^36^

### Digital image acquisition

The TMAs were comprised of three tumor cores and three cores of adjacent normal stroma for each patient. Following immunohistochemical staining, each TMA was digitally scanned at high resolution in the Digital Pathology Laboratory, Northern Ireland Molecular Pathology Laboratory within the Centre for Cancer Research and Cell Biology, Queen’s University, Belfast, UK. The slides were scanned using an Aperio ScanScope CS (Aperio Technologies, San Diego, CA, USA) at a resolution of 40× using the objective 40×/0.75 Plan Apo with a doubler and loaded onto the local drive for storage.

### Statistical analyses

To define H-score cutoff values, we adopted the approach described by Zlobec *et al.*^37^ for using the receiver operating characteristic (ROC). Using R statistical environment version 2.3.3, we employed the pROC software package to determine the sensitivity and specificity of H-score cutoff values for each marker (Tables 2 and 3), with subsequent validation of the most discriminatory cutoffs assessed using Kaplan–Meier curves derived using the survival package within pROC (R Core Team: https://www.r-project.org/^38^).

Descriptive characteristics and overall survival of patients were evaluated according to histological subtype of lung tumor (adenocarcinoma or squamous cell carcinoma). Cox proportional hazards analysis was conducted exploring risk of death in lung adenocarcinoma or squamous cell carcinoma patients, according to their expression levels of FLIP and procaspase-8 in the tumoral nucleus or cytoplasm or the stromal nucleus or cytoplasm. Analysis was conducted prior to and after adjustment for potential confounders, such as age, sex, smoking status, tumor stage, tumor grade, tumor size and extent of surgical resection conducted. Follow-up was defined as the date of surgery up to the date of death or last known date of vital status. To explore the influence of early deaths in the associations observed, sensitivity analysis was conducted after removing lung cancer patients who died within the first 6 months of follow-up. Cox proportional hazards analysis was conducted in Stata Version 11.2 (StataCorp, College Station, TX, USA). Based on *a priori* biological plausibility, stratified analysis of risk of death in combined categories of high and low cytoplasmic FLIP and procaspase-8 expression was evaluated.

## Publisher’s note

Springer Nature remains neutral with regard to jurisdictional claims in published maps and institutional affiliations.

## Figures and Tables

**Table 1 tbl1:** Characteristics of lung cancer patients according to histological subtype

*Characteristic*	*Adenocarcinoma,* n*=86*	*Squamous cell carcinoma,* n=*89*
Age, years, median (range)	65.7 (42.5–82.7)	66.0 (41.4–86.2)
		
*Sex*, n *(%)*		
Male	46 (53.5)	63 (70.8)
Female	40 (46.5)	26 (29.2)
		
*Tumor stage*, n *(%)*		
I	49 (57.0)	46 (51.7)
II	14 (16.3)	26 (29.2)
III	23 (26.7)	17 (19.1)
		
*Tumor grade*, n *(%)*		
I	9 (10.5)	2 (2.2)
II	51 (59.3)	55 (61.8)
III	26 (30.2)	32 (36.0)
		
Tumor size, cm, median (range)	3.8 (1–14)	4.0 (0.9–16)
		
*Smoking status*		
Current	26 (30.2)	26 (29.2)
Former	53 (61.6)	60 (67.4)
Never	7 (8.1)	3 (3.4)
		
*Surgery type*, n *(%)*		
Lobectomy	69 (80.2)	60 (67.4)
Bilobectomy	10 (11.6)	4 (4.5)
Pneumonectomy	7 (8.1)	25 (28.1)
		
*Vital status by end of follow-up*, n *(%)*		
Alive	35 (40.7)	37 (41.6)
Dead	51 (59.3)	52 (58.4)

**Table 2:** Risk of death in lung adenocarcinoma patients according to FLIP and procaspase-8 expression within the nucleus and cytoplasm in tumor and stromal compartments

	*Alive,* n*=35 (%)*	*Dead,* n*=51 (%)*	*Unadjusted hazard ratios (95% confidence intervals)*	*Adjusted*^a^ *hazard ratios (95% confidence intervals)*
*Tumoral FLIP nucleus*				
Low (⩽105)	24 (68.6)	27 (52.9)	1.00	1.00
High (>105)	11 (31.4)	24 (47.1)	1.62 (0.93–2.81)	1.50 (0.81–2.81)
				
*Tumoral FLIP cytoplasm*				
Low (⩽172.5)	15 (42.9)	11 (21.6)	1.00	1.00
High (>172.5)	20 (57.1)	40 (78.4)	1.97 (1.01–3.84)	2.47 (1.14–5.35)
				
*Stromal FLIP nucleus*				
Low (⩽74.5)	18 (51.4)	31 (60.8)	1.00	1.00
High (>74.5)	17 (48.6)	20 (39.2)	0.79 (0.45–1.39)	0.75 (0.40–1.41)
				
*Stromal FLIP cytoplasm*				
Low (⩽144.5)	17 (48.6)	33 (64.7)	1.00	1.00
High (>144.5)	18 (51.4)	18 (35.3)	0.65 (0.36–1.15)	0.57 (0.30–1.08)
				
*Tumoral procaspase-8 nucleus*				
Low (⩽73.5)	6 (17.1)	16 (31.4)	1.00	1.00
High (>73.5)	29 (82.9)	35 (68.6)	0.65 (0.36–1.18)	0.94 (0.40–2.19)
				
*Tumoral procaspase-8 cytoplasm*				
Low (⩽155.5)	14 (40.0)	16 (31.4)	1.00	1.00
High (>155.5)	21 (60.0)	35 (68.6)	1.25 (0.69–2.27)	1.57 (0.75–3.31)
				
*Stromal procaspase-8 nucleus*				
Low (⩽90.5)	15 (42.9)	39 (76.5)	1.00	1.00
High (>90.5)	20 (57.1)	12 (23.5)	0.40 (0.21–0.76)	0.31 (0.15–0.66)
				
*Stromal procaspase-8 cytoplasm*				
Low (⩽139.5)	22 (62.9)	42 (82.4)	1.00	1.00
High (>139.5)	13 (37.1)	9 (17.7)	0.51 (0.25–1.05)	0.51 (0.23–1.11)

a Adjustments included age, sex, smoking status, TNM stage, tumor size, tumor grade and surgery type.

**Table 3:** Risk of death in lung squamous cell carcinoma patients according to FLIP and procaspase-8 expression within the nucleus and cytoplasm in tumor and stromal compartments

	*Alive,* n*=37 (%)*	*Dead,* n*=52 (%)*	*Unadjusted hazard ratios (95% confidence intervals)*	*Adjusted*^a^ *hazard ratios (95% confidence intervals)*
*Tumoral FLIP nucleus*				
Low (⩽92)	7 (18.9)	19 (36.5)	1.00	1.00
High (>92)	30 (81.1)	33 (63.5)	0.70 (0.40–1.23)	0.66 (0.36–1.23)
				
*Tumoral FLIP cytoplasm*				
Low (⩽231)	28 (75.7)	28 (53.9)	1.00	1.00
High (>231)	9 (24.3)	24 (46.2)	1.62 (0.94–2.81)	1.31 (0.70–2.44)
				
*Stromal FLIP nucleus*				
Low (⩽86.5)	22 (59.5)	41 (78.9)	1.00	1.00
High (>86.5)	15 (40.5)	11 (21.2)	0.55 (0.28–1.06)	0.52 (0.25–1.10)
				
*Stromal FLIP cytoplasm*				
Low (⩽127.5)	18 (48.7)	17 (32.7)	1.00	1.00
High (>127.5)	19 (51.4)	35 (67.3)	1.41 (0.79–2.51)	1.08 (0.57–2.05)
				
*Tumoral procaspase-8 nucleus*				
Low (⩽59.5)	8 (21.6)	17 (32.7)	1.00	1.00
High (>59.5)	29 (78.4)	35 (67.3)	0.77 (0.43–1.38)	0.73 (0.37–1.44)
				
*Tumoral procaspase-8 cytoplasm*				
Low (⩽147.5)	10 (27.0)	21 (40.4)	1.00	1.00
High (>147.5)	27 (73.0)	31 (59.6)	0.87 (0.50–1.52)	0.84 (0.45–1.55)
				
*Stromal procaspase-8 nucleus*				
Low (⩽36.5)	16 (43.2)	16 (30.8)	1.00	1.00
High (>36.5)	21 (56.8)	36 (69.2)	1.49 (0.83–2.70)	1.64 (0.87–3.09)
				
*Stromal procaspase-8 cytoplasm*				
Low (⩽88.5)	11 (29.7)	19 (36.5)	1.00	1.00
High (>88.5)	26 (70.3)	33 (63.5)	0.98 (0.56–1.73)	1.16 (0.59–2.28)

a Adjustments included age, sex, smoking status, TNM stage, tumor size, tumor grade and surgery type.
